# Supplementary material for: Rescuing the bacterial replisome at a nick requires recombinational repair and helicase reloading
Source: Nat Commun. 2025 Nov 26;16:11633. doi: 10.1038/s41467-025-66550-w (PMC12748557; doi:10.1038/s41467-025-66550-w)
Supplement: Supplementary file 1 — Supplementary Information [file 41467_2025_66550_MOESM1_ESM.pdf]

SUPPLEMENTARY INFORMATION

TITLE

Rescuing the bacterial replisome at a nick requires recombinational repair and helicase reloading

AUTHOR LIST

Charles Winterhalter[1*], Kathryn J Stratton[1], Stepan Fenyk[1], Heath Murray[1*]

AFFILIATION

[1]Centre for Bacterial Cell Biology, Biosciences Institute, Newcastle University, Newcastle Upon Tyne, NE2 4AX, UK

*Correspondence to: Charles.winterhalter@newcastle.ac.uk
Heath.murray@newcastle.ac.uk

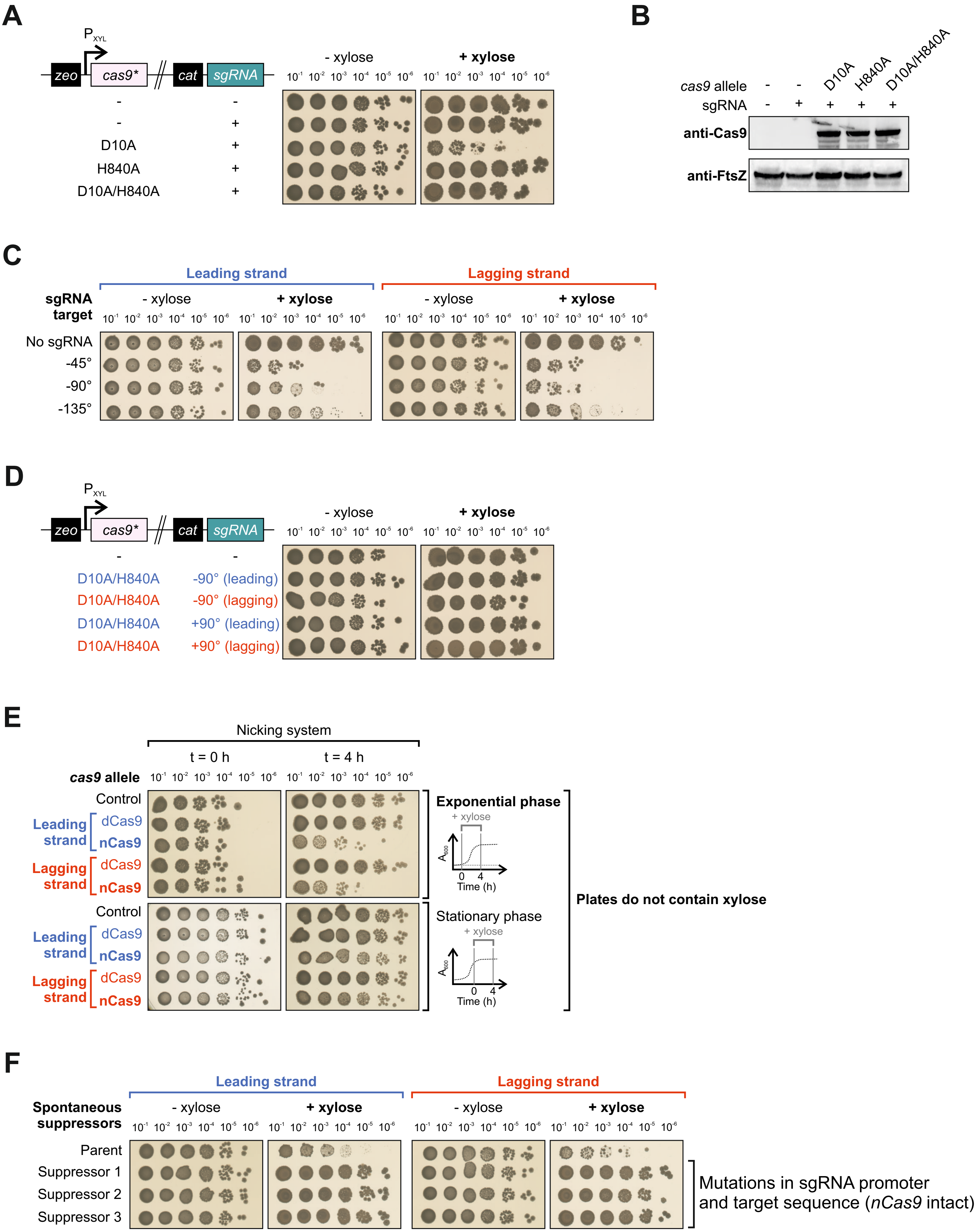

SUPPLEMENTARY FIGURE 1

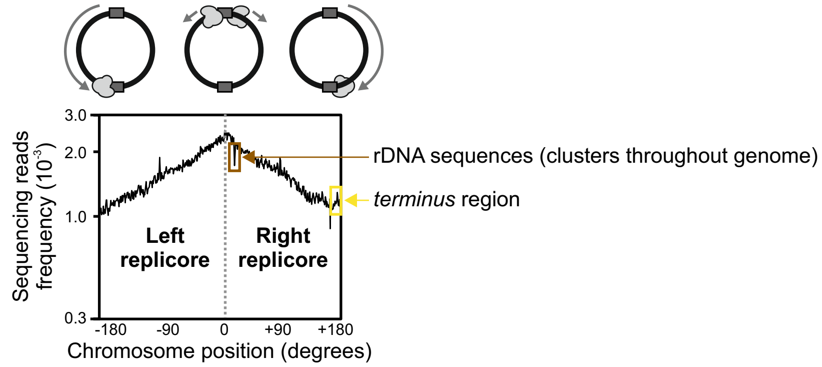

SUPPLEMENTARY FIGURE 2

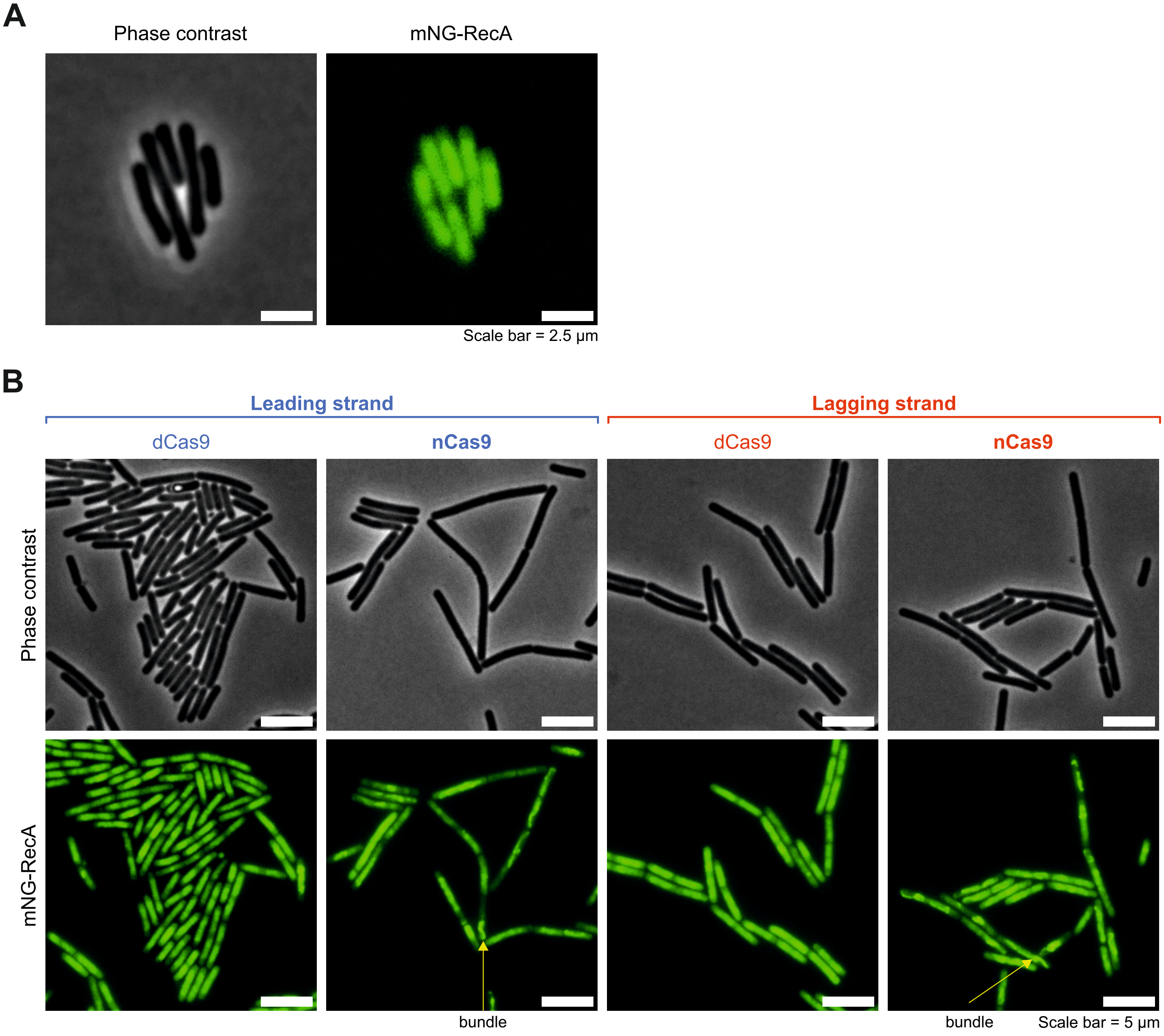

SUPPLEMENTARY FIGURE 3

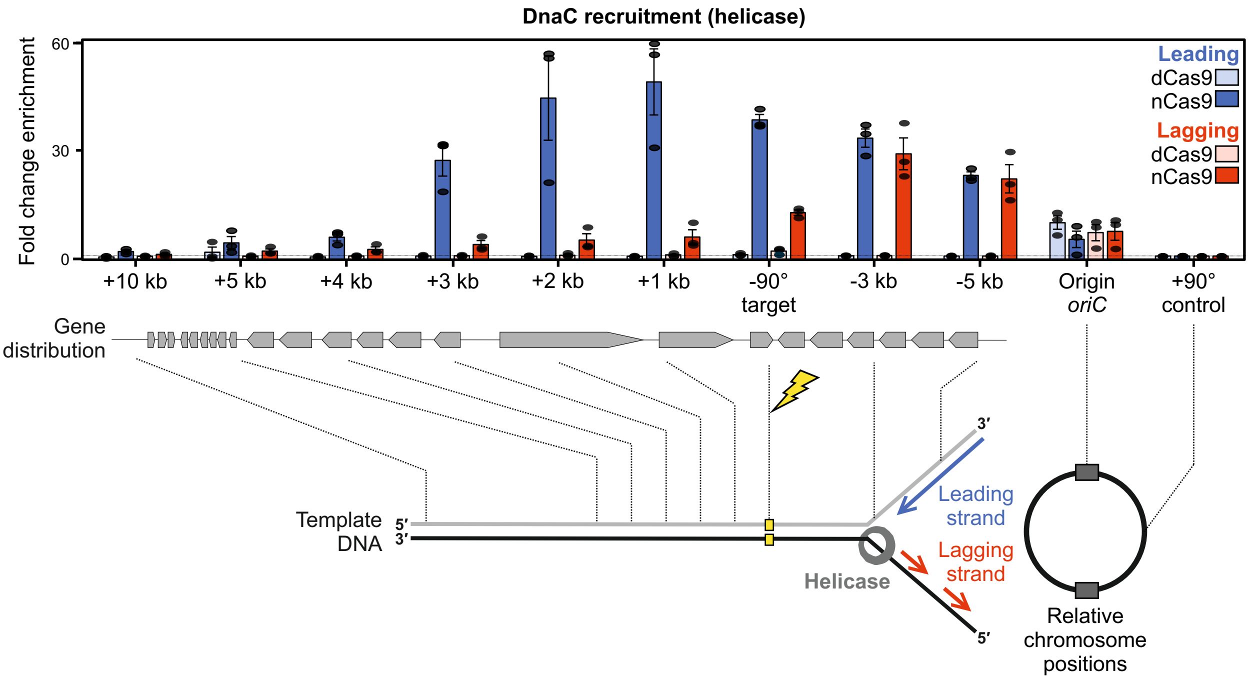

SUPPLEMENTARY FIGURE 4

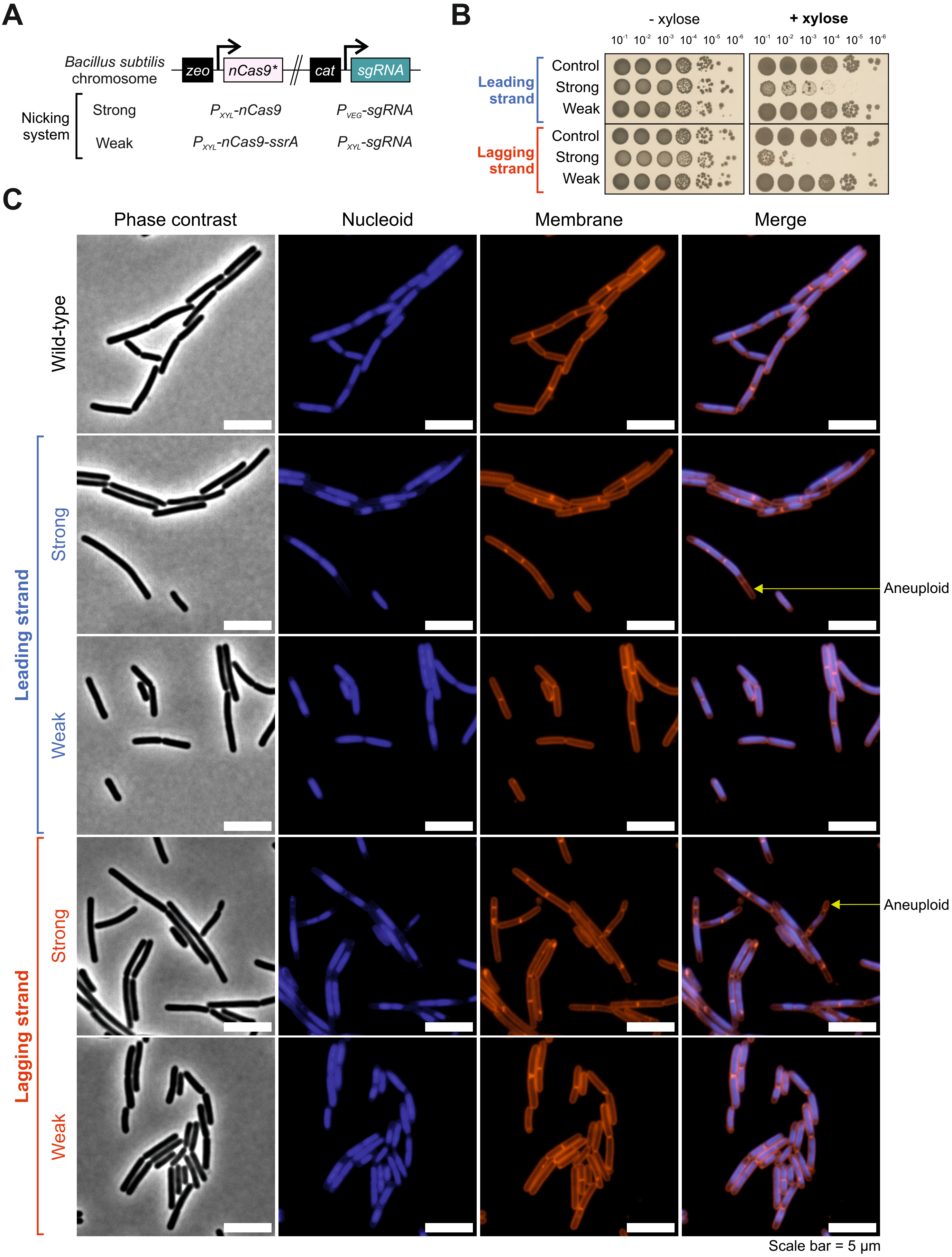

SUPPLEMENTARY FIGURE 5

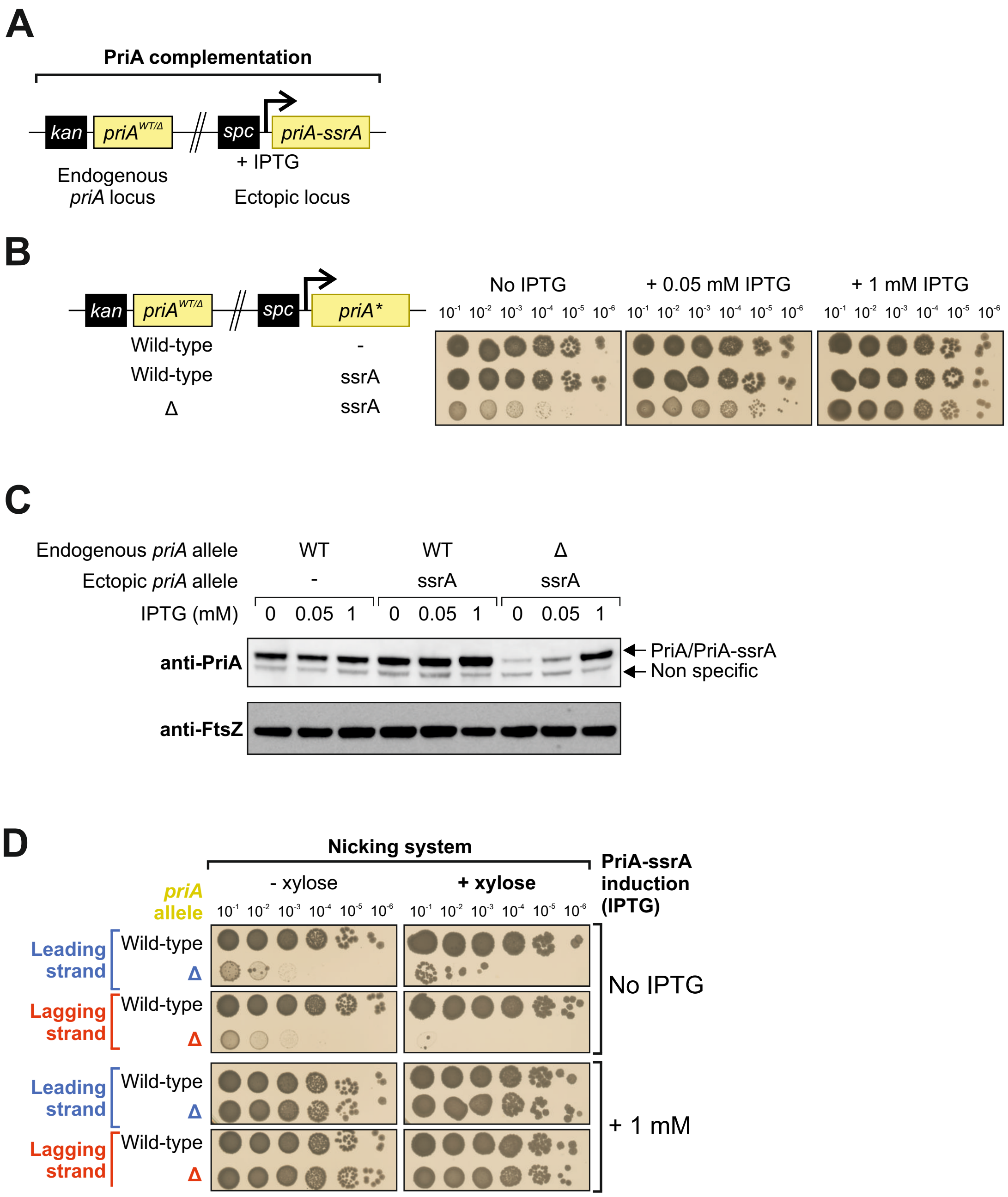

SUPPLEMENTARY FIGURE 6

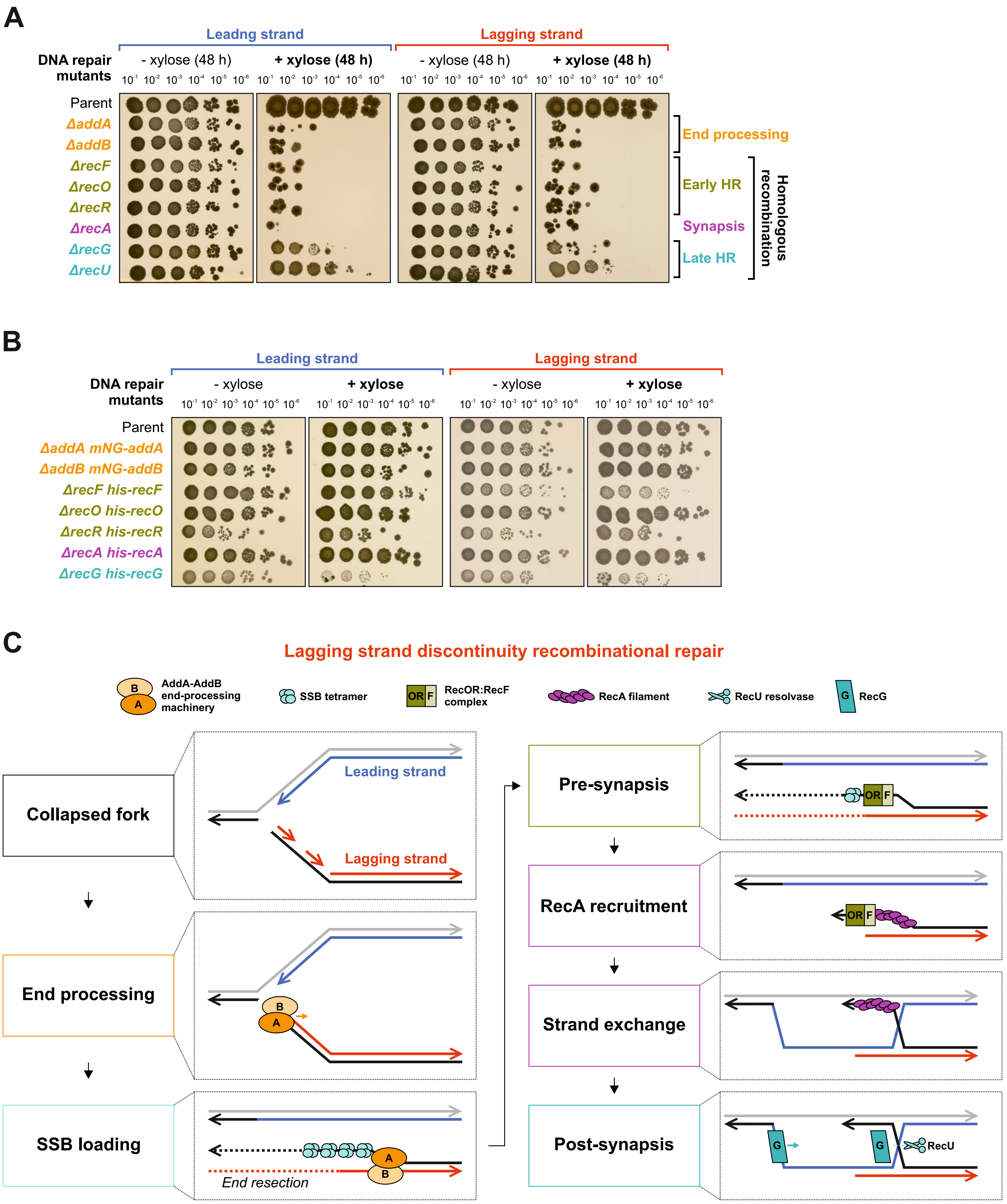

SUPPLEMENTARY FIGURE 7

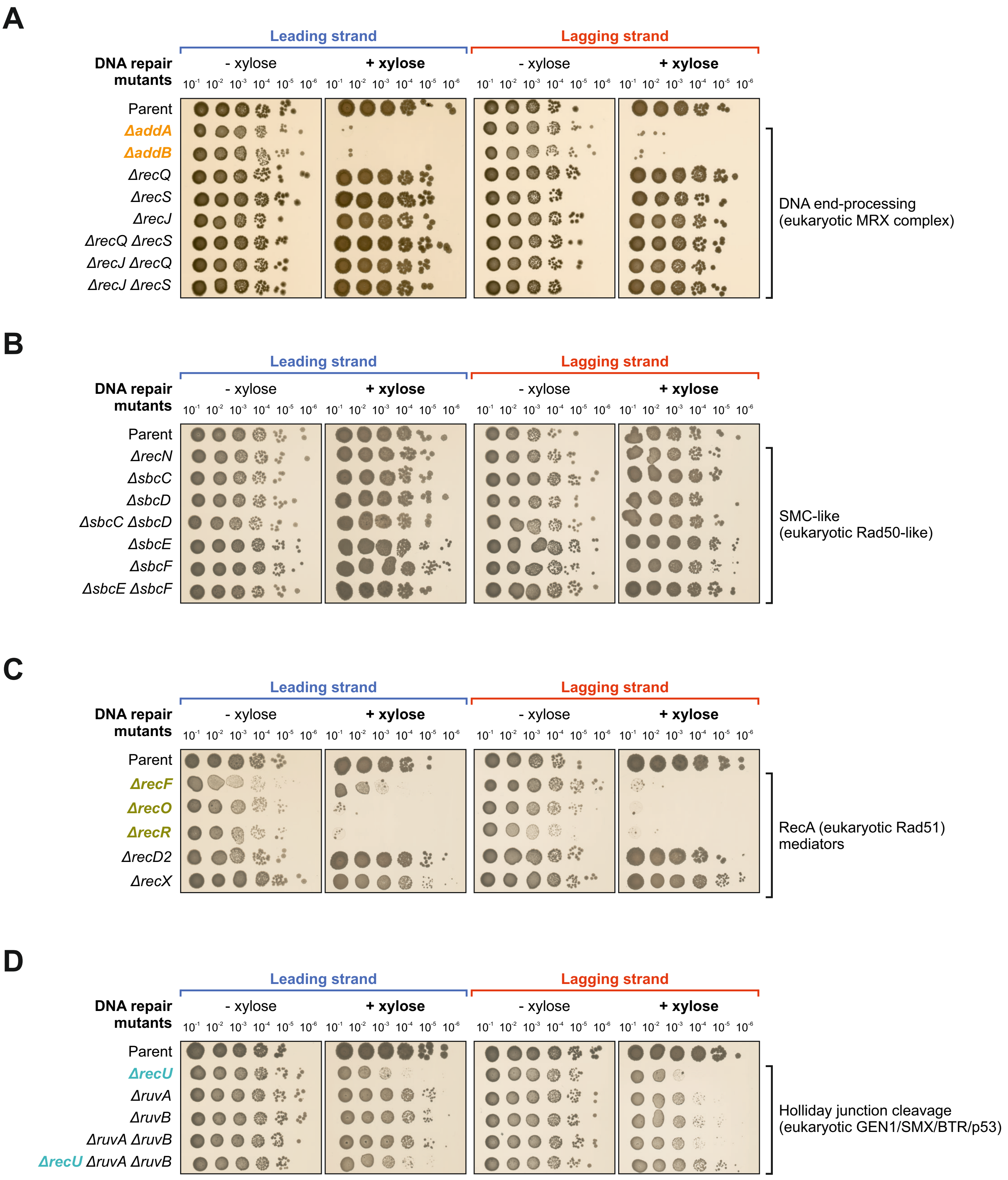

SUPPLEMENTARY FIGURE 8

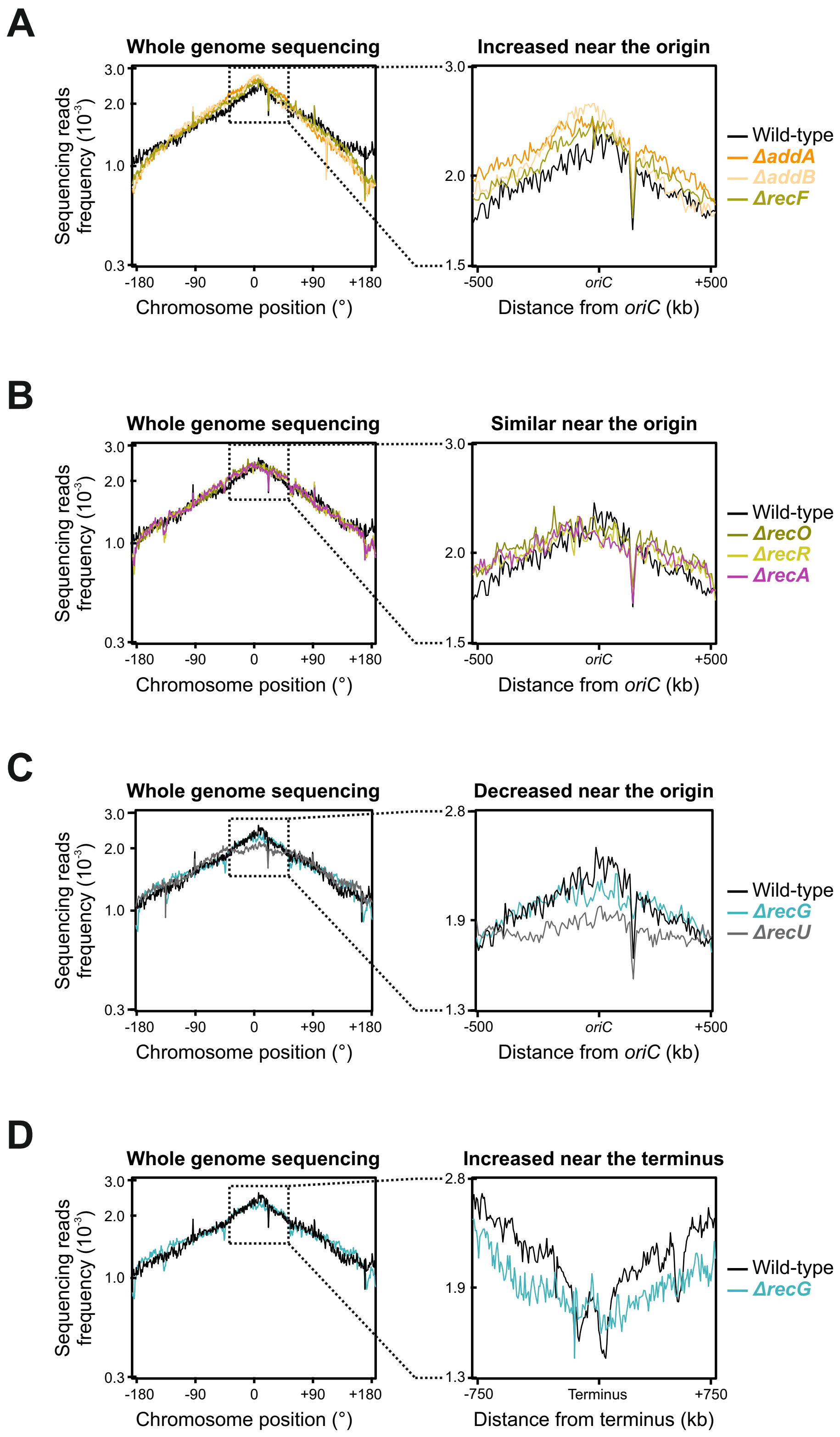

SUPPLEMENTARY FIGURE 9

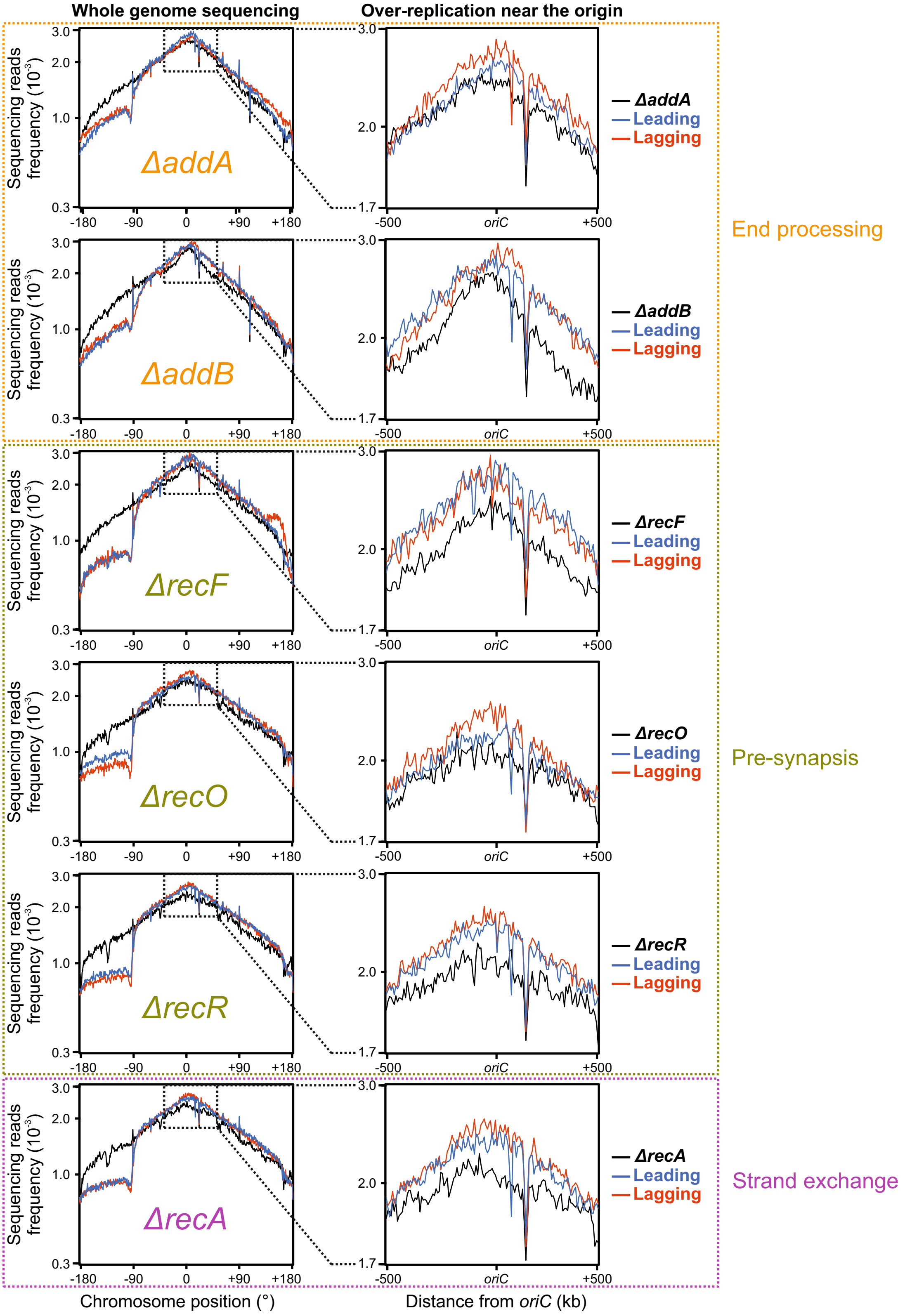

SUPPLEMENTARY FIGURE 10

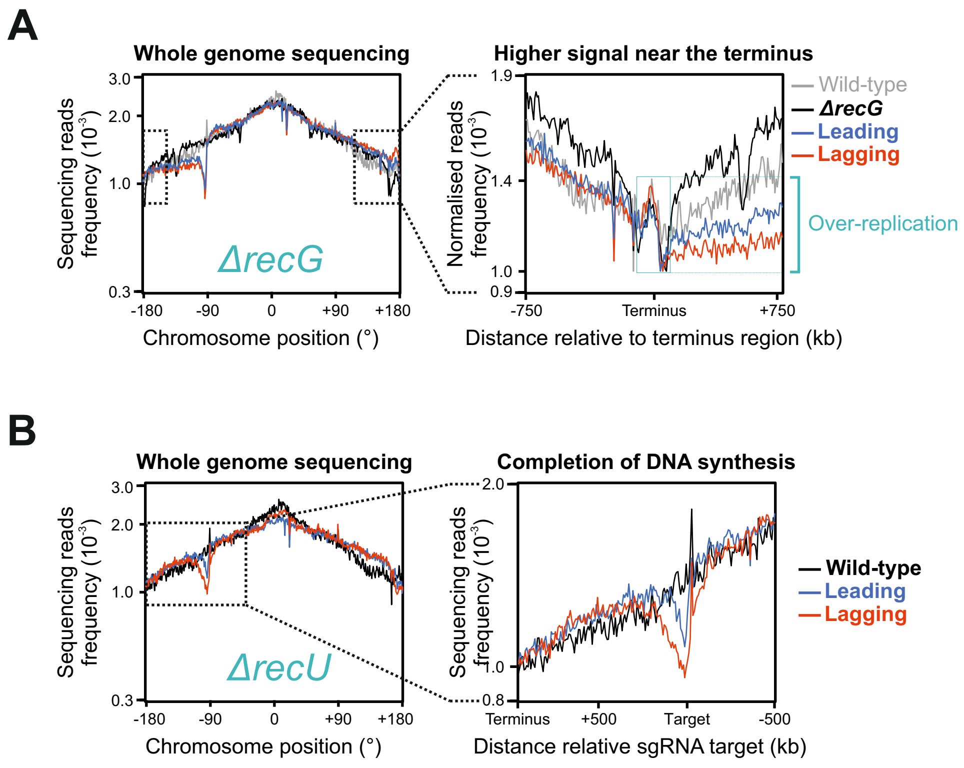

SUPPLEMENTARY FIGURE 11

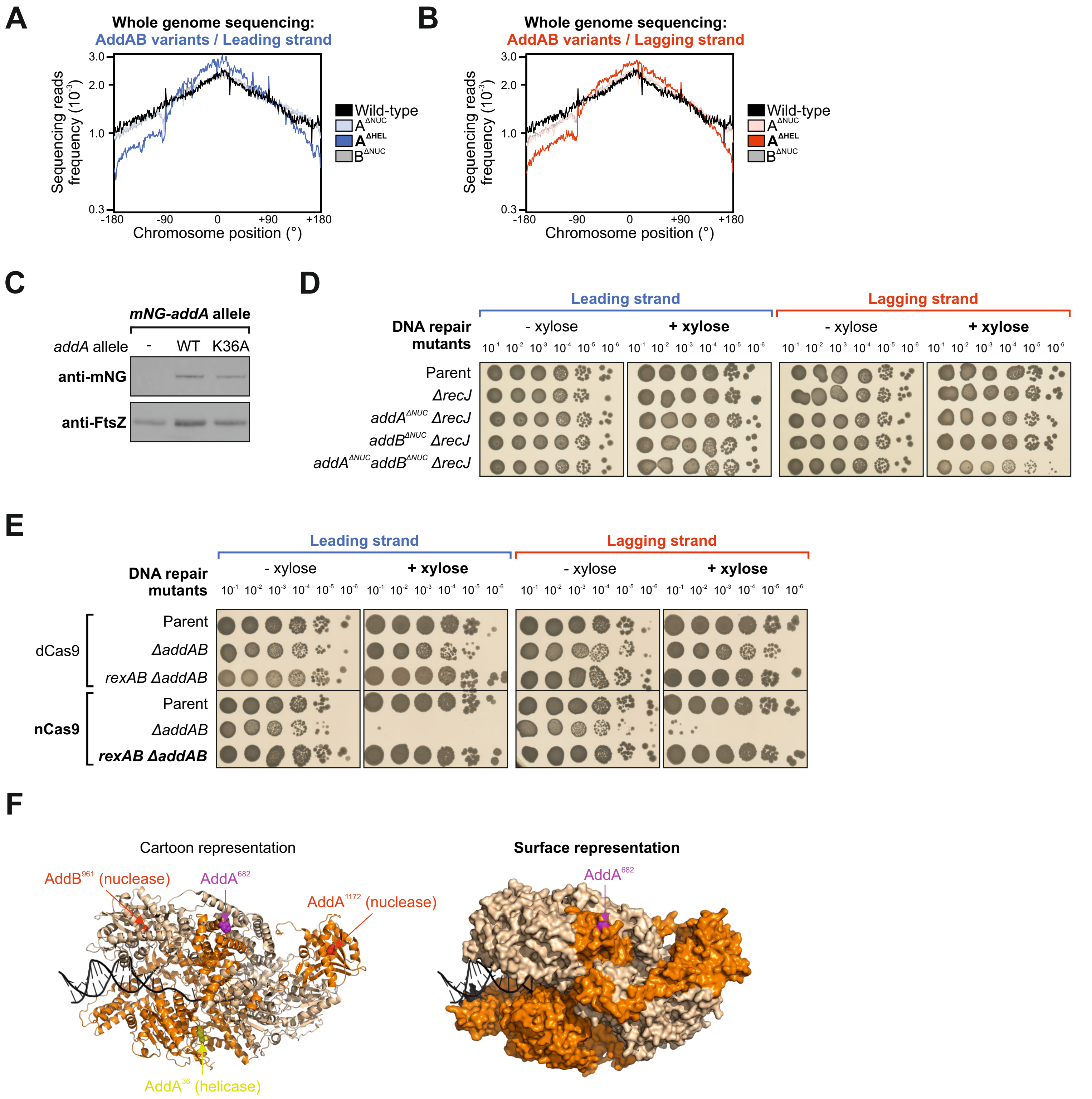

SUPPLEMENTARY FIGURE 12

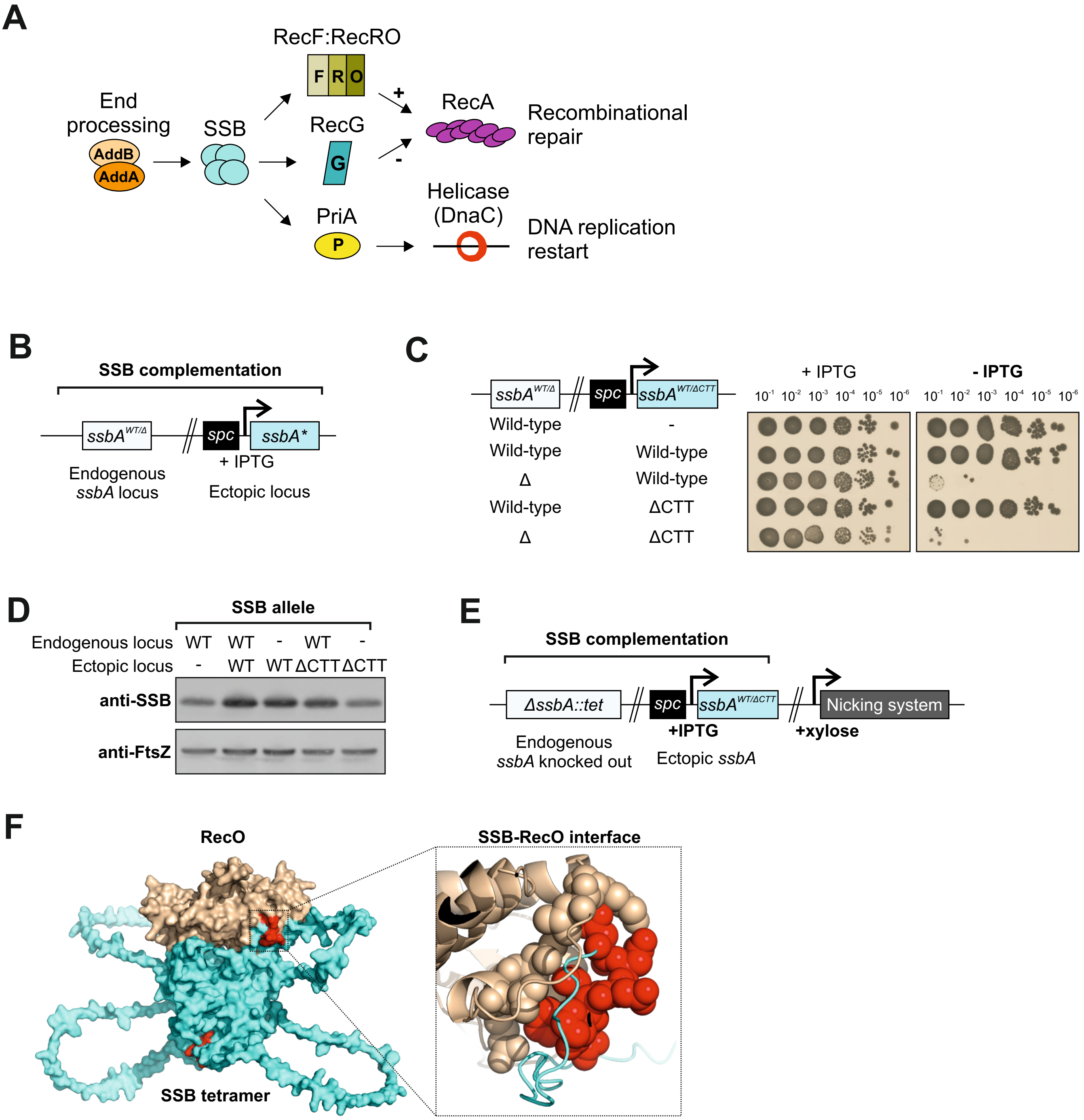

SUPPLEMENTARY FIGURE 13

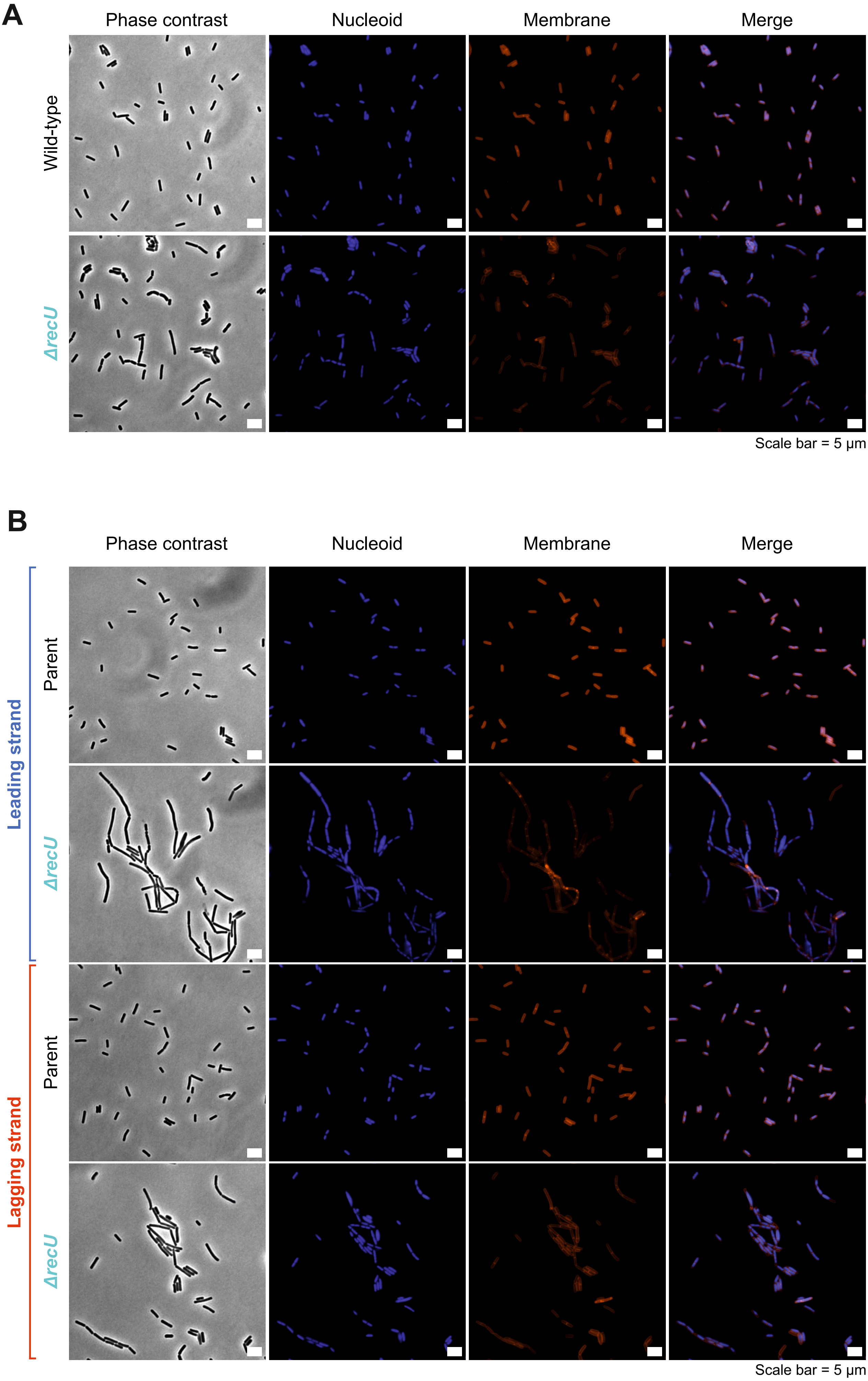

SUPPLEMENTARY FIGURE 14

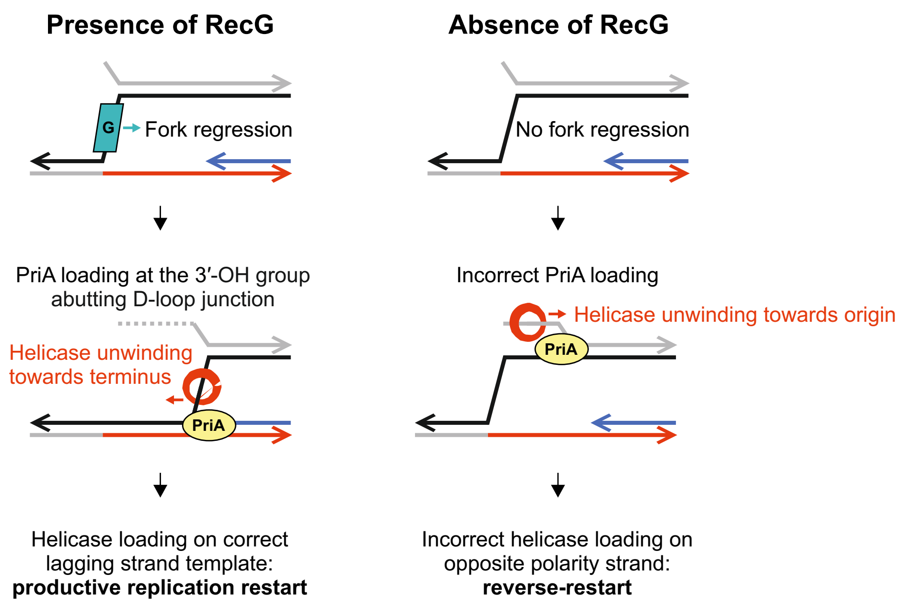

SUPPLEMENTARY FIGURE 15

SUPPLEMENTARY FIGURE LEGENDS/CAPTIONS

Supplementary Figure 1. Impact of Cas9 variants on *B. subtilis* growth.

(A) Spot-titre analysis of strains expressing (+xylose) either $Cas9^{D10A}$ nickase, $Cas9^{H840A}$ nickase, or $Cas9^{D10A/H840A}$ catalytically inactive nuclease in the presence of the same sgRNA. Left panel shows the genetic system employed in this experiment. sgRNAs were targeted to a locus at -90°.

(B) Immunoblot of Cas9 variants in the presence of a sgRNA targeted to a locus at -90°. Cas9 proteins were induced (+xylose) during the exponential growth phase for 1 hour before cells were harvested for protein extraction. The tubulin homolog FtsZ was used as a loading control and data is representative from at least two biological repeats.

(C) Spot-titre analysis of a strains expressing (+xylose) the $Cas9^{D10A}$ nickase with different sgRNAs targeting locations along the left chromosome arm.

(D) Spot-titre analysis of strains expressing (+xylose) the $Cas9^{D10A\text{-}H840A}$ catalytically inactive nuclease. Left panel shows the genetic system employed in this experiment for sgRNAs targeted to a locus at either -90° or +90°.

(E) Strains were induced to express (+xylose) nCas9 or dCas9 during exponential or stationary phase for 4 hours, followed by spot-titre analysis in the absence of xylose to determine CFUs remaining after treatment. sgRNAs were targeted to a locus at -90°.

(F) Spot-titre analysis of suppressor colonies isolated from strains expressing (+xylose) nCas9. sgRNAs were targeted to a locus at -90°. The parental nicking strain control is shown above. Identity of suppressors was identified via sequencing.

Source data are provided as a Source Data file.

Supplementary Figure 2. MFA using whole genome sequencing of *B. subtilis* Δ6.

The frequency of sequencing reads was plotted against genome position. Note that arbitrary chromosome boundaries indicated on x-axis may not represent termination events occurring in the cell (*terminus* region highlighted in yellow). Redundant ribosomal DNA sequences result in peaks throughout the genome (rDNA sequences highlighted in brown).

Source data are provided as a Source Data file.

Supplementary Figure 3. Live cell fluorescence microscopy using the mNG-RecA DNA damage reporter.

(A) Phase contrast image and fluorescence signal from the mNG-RecA reporter in wild-type *B. subtilis* under standard laboratory growth conditions. Scale bar indicates 2.5 µm.

(B) Fluorescence microscopy images of mNG-RecA in strains engineered to express either nCas9 or dCas9 (sgRNA target -90°). Fluorescent RecA organises into bundles in the presence of DNA damage. Green signal corresponds to mNG-RecA fluorescence, grey scale images show corresponding phase contrast images. Cas9 proteins were induced (+xylose) during the exponential growth phase for 1 hour before cells were collected for imaging. Scale bar indicates 5 µm.

Source data are provided as a Source Data file.

Supplementary Figure 4. The replicative helicase DnaC is recruited near a single-strand discontinuity.

ChIP-qPCR analyses of helicase (DnaC) in strains engineered to express either nCas9 or dCas9. The sgRNAs targeted Cas9 proteins to a locus located at -90°. The replication origin (*oriC*) was used as positive control and protein enrichment was normalised to the control locus at +90°. Error bars indicate the standard error of the mean and circles overlaid on bars correspond to three biological replicates.

Source data are provided as a Source Data file.

Supplementary Figure 5. An attenuated nCas9 system does not arrest cell growth.

(A) Schematic showing differences between the strong and weak nicking systems used to introduce a single-strand discontinuity.

(B) Spot-titre analysis of strains expressing (+xylose) the strong or weak nicking system in the presence of the same sgRNA. Control indicates a strain encoding the Cas9$^{D10A/H840A}$ catalytically inactive nuclease. sgRNAs targeted to a locus located at -90°.

(C) Live cell fluorescence microscopy in strains expressing either the strong or the weak nCas9 system. Grey scale images correspond to phase contrast, blue signal corresponds to DAPI staining (nucleoid), red signal corresponds to Nile red staining (membrane) and merge corresponds to DAPI/Nile red composite images. Cas9 proteins were induced (+xylose) during the exponential growth phase for 90 minutes before cells were collected for imaging.

Source data are provided as a Source Data file.

Supplementary Figure 6. System employed to limit PriA expression during replisome inactivation at single-strand discontinuities.

(A) Genetic system employed for *priA* complementation. The IPTG inducible *priA-ssrA* cassette was placed at an ectopic locus.

(B) Spot-titre analysis of strains expressing different levels of PriA-ssrA (IPTG-dependent) in the presence or absence of the endogenous *priA*.

(C) Immunoblot of PriA variants expressed at a range of IPTG concentrations. The tubulin homolog FtsZ was used as a loading control and data is representative from at least two biological repeats.

(D) Spot-titre analysis of strains with a low or high level of PriA-ssrA (uninduced or 1 mM IPTG) engineered to express nCas9 (+xylose). sgRNAs were targeted to a locus at -90°.

Source data are provided as a Source Data file.

Supplementary Figure 7. Recombinational repair is necessary for viability following replisome inactivation at a single-strand discontinuity.

(A) Spot-titre analysis of recombinational repair mutants in strains engineered to express (+xylose) nCas9. sgRNAs were targeted to a locus at -90°. The parental nicking strain control is shown above. Plates were the same as shown in Fig. 3A imaged after 48 hours.

(B) Spot-titre analysis of recombinational repair mutants complemented with ectopic epitope-tagged copies in strains engineered to express (+xylose) nCas9. sgRNAs were targeted to a locus at -90°. The parental weak nicking strain is shown above.

(C) Diagram illustrating proposed molecular events required for recombinational repair to repair the seDSB created after a replication fork encounters a single-strand discontinuity in the lagging strand template.

Supplementary Figure 8. Viability following replisome inactivation at a single-strand discontinuity relies on a minimal set of recombinational repair genes.

(A-D) Spot-titre analysis of DNA repair mutants grouped by known/proposed functions in strains engineered to express (+xylose) nCas9. sgRNAs were targeted to a locus at -90°. The parental nicking strain control is shown above.

Supplementary Figure 9. MFA using whole genome sequencing of recombinational repair mutants.

(A-C) MFA using whole genome sequencing of (A) Δ*addA*, Δ*addB,* Δ*recF*, (B) Δ*recO*, Δ*recR,* Δ*recA*, (C) Δ*recU*, and (C-D) Δ*recG* mutant strains. The frequency of sequencing reads was plotted against genome position (same data as in Fig. 3B for background). A region flanking the replication origin is enlarged.

Source data are provided as a Source Data file.

Supplementary Figure 10. MFA using whole genome sequencing of recombinational repair mutants in strains expressing nCas9: highlighting the *oriC* region.

MFA using whole genome sequencing of recombinational repair mutant strains engineered to express nCas9. The frequency of sequencing reads was plotted against genome position (same data as in Fig. 3B knockout panels). A region flanking the replication origin is enlarged.

Source data are provided as a Source Data file.

Supplementary Figure 11. MFA using whole genome sequencing of post-synapsis recombinational repair mutants in strains expressing nCas9.

(A-B) MFA using whole genome sequencing in either (A) Δ*recG* or (A) Δ*recU* mutant strains engineered to express nCas9. The frequency of sequencing reads was plotted against genome position (same data as in Fig. 3B post-synapsis panels). Genomic regions of interest are enlarged.

Source data are provided as a Source Data file.

Supplementary Figure 12. End-processing activities required following replisome inactivation at a single-strand discontinuity.

(A-B) MFA using whole genome sequencing of strains with either $AddA^{\Delta HEL}$, $AddA^{\Delta NUC}$ or $AddB^{\Delta NUC}$ engineered to express nCas9. The frequency of sequencing reads was plotted against genome position. $AddA^{\Delta HEL}$ samples were the same as shown in Fig. 4D.

(C) Immunoblot of mNG-AddA variants. The tubulin homolog FtsZ was used as a loading control and data is representative from at least two biological repeats.

(D) Spot-titre analysis of Δ*recJ* mutants combined with AddAB nuclease deficient variants in strains engineered to express (+xylose) nCas9. sgRNAs were targeted to a locus at -90°. The parental nicking strain control is shown above.

(E) Spot-titre analysis of Δ*addAB* mutants complemented with *rexAB* in strains engineered to express (+xylose) nCas9. sgRNAs were targeted to a locus at -90°. The parental nicking strain control is shown above.

(F) Crystal structure of the *B. subtilis* AddAB complex with locations of catalytic sites and the $AddA^{D682}$ residue indicated (PDB template: 4CEH). The black backbone corresponds to DNA, AddA is displayed in orange and AddB in beige.

Source data are provided as a Source Data file.

Supplementary Figure 13. System employed to investigate the function of the SSB-CTT following replisome inactivation at single-strand discontinuities.

(A) Diagram illustrating potential essential interactions of the SSB-CTT.

(B) Genetic system employed for *ssbA* complementation. An IPTG inducible copy of *ssbA* was placed at an ectopic locus.

(C) Spot-titre analysis of strains expressing (+IPTG) *ssbA* variants with and without the endogenous *ssbA*.

(D) Immunoblot of SSB variants expressed using the *ssbA* complementation system. The tubulin homolog FtsZ was used as a loading control and data is representative from at least two biological repeats.

(E) Genetic system employed to express SSB variants in a background encoding the weak nicking system.

(F) Structural model of a complex between a SSB homotetramer and RecO from *B. subtilis* (AlphaFold 3). Cyan corresponds to SSB with CTT residues coloured in red and RecO is displayed in beige.

Source data are provided as a Source Data file.

Supplementary Figure 14. A Δ*recU* mutant displays chromosome segregation defects.

(A-B) Live cell fluorescence microscopy of Δ*recU* mutants using nucleoid and membrane stains in (A) wild-type or (B) strains expressing nCas9. Grey scale images correspond to phase contrast, blue signal corresponds to DAPI staining (nucleoid), red signal corresponds to Nile red staining (membrane) and merge corresponds to DAPI/Nile red composite images. Cas9 proteins were induced (+xylose) during the exponential growth phase for 90 minutes before cells were collected for imaging.

Source data are provided as a Source Data file.

Supplementary Figure 15. Potential role of RecG in replication fork remodelling.

Diagram illustrating the proposed function of RecG to ensure productive helicase reloading following replisome inactivation at a single-strand discontinuity[1].

SUPPLEMENTARY REFERENCES

1. Azeroglu B, *et al*. RecG Directs DNA Synthesis during Double-Strand Break Repair. *PLoS Genet* **12**, e1005799 (2016).
