# Supplementary material for: Rescuing the bacterial replisome at a nick requires recombinational repair and helicase reloading
Source: Nat Commun. 2025 Nov 26;16:11633. doi: 10.1038/s41467-025-66550-w (PMC12748557; doi:10.1038/s41467-025-66550-w)
Supplement: Supplementary file 2 — Description of Additional Supplementary Files [file 41467_2025_66550_MOESM2_ESM.pdf]

Description of Additional Supplementary Files

File name: Supplementary Data 1

Description: List of repair gene knockouts tested in the genetic screen (related to Fig. 3 and Supplementary Figure 8)

File Name: Supplementary Data 2

Description: List of genotypes from strains generated in this study.

File Name: Supplementary Data 3

Description: List of genotypes and sequences of recombinant DNA used to construct strains in this study, related to Supplementary Data 2.

File Name: Supplementary Data 4

Description: List of oligonucleotide sequences used for qPCR in this study.
