# Supplementary material for: Rescuing the bacterial replisome at a nick requires recombinational repair and helicase reloading
Source: Nat Commun. 2025 Nov 26;16:11633. doi: 10.1038/s41467-025-66550-w (PMC12748557; doi:10.1038/s41467-025-66550-w)
Supplement: Supplementary file 4 — Reporting Summary [file 41467_2025_66550_MOESM4_ESM.pdf]

nature portfolio

Corresponding author(s): Charles Winterhalter, Heath Murray

Last updated by author(s): Oct 23, 2025

Reporting Summary

Nature Portfolio wishes to improve the reproducibility of the work that we publish. This form provides structure for consistency and transparency in reporting. For further information on Nature Portfolio policies, see our Editorial Policies and the Editorial Policy Checklist.

Statistics

For all statistical analyses, confirm that the following items are present in the figure legend, table legend, main text, or Methods section.

n/a	Confirmed	
☐	☒	The exact sample size (*n*) for each experimental group/condition, given as a discrete number and unit of measurement
☐	☒	A statement on whether measurements were taken from distinct samples or whether the same sample was measured repeatedly
☐	☒	The statistical test(s) used AND whether they are one- or two-sided<br>*Only common tests should be described solely by name; describe more complex techniques in the Methods section.*
☐	☒	A description of all covariates tested
☒	☐	A description of any assumptions or corrections, such as tests of normality and adjustment for multiple comparisons
☐	☒	A full description of the statistical parameters including central tendency (e.g. means) or other basic estimates (e.g. regression coefficient) AND variation (e.g. standard deviation) or associated estimates of uncertainty (e.g. confidence intervals)
☐	☒	For null hypothesis testing, the test statistic (e.g. *F*, *t*, *r*) with confidence intervals, effect sizes, degrees of freedom and *P* value noted<br>*Give P values as exact values whenever suitable.*
☒	☐	For Bayesian analysis, information on the choice of priors and Markov chain Monte Carlo settings
☒	☐	For hierarchical and complex designs, identification of the appropriate level for tests and full reporting of outcomes
☒	☐	Estimates of effect sizes (e.g. Cohen's *d*, Pearson's *r*), indicating how they were calculated

Our web collection on statistics for biologists contains articles on many of the points above.

Software and code

Policy information about availability of computer code

Data collection	All data collection is clearly described in the manuscript.<br>QIAGEN CLC Genomics Workbench (version 23) was used to generate .BAM files from whole-genome sequencing data. R (version 4.5) was used to process .BAM files. NIS-Elements (version 5) and Fiji (version 1.53) were used for microscopy data acquisition and analyses, respectively.
Data analysis	All data analyses are clearly described in the manuscript.<br>CODE AVAILABILITY STATEMENT. The R code used to process next-generation sequencing data used in this study is available under open access (CC BY) in the Zenodo database under accession code DOI: 10.5281/zenodo.14793122.

For manuscripts utilizing custom algorithms or software that are central to the research but not yet described in published literature, software must be made available to editors and reviewers. We strongly encourage code deposition in a community repository (e.g. GitHub). See the Nature Portfolio guidelines for submitting code & software for further information.

Data

Policy information about availability of data

All manuscripts must include a data availability statement. This statement should provide the following information, where applicable:

- Accession codes, unique identifiers, or web links for publicly available datasets
- A description of any restrictions on data availability
- For clinical datasets or third party data, please ensure that the statement adheres to our policy

DATA AVAILABILITY STATEMENT. Source data generated in this study are provided with this paper on Figshare. The SSB:ssDNA and SSB:RecO models generated by AlphaFold 3 in this study have been deposited in the Zenodo database under accession code DOI: 10.5281/zenodo.14793919. All data presented in this study are provided in the Supplementary Information/Source Data file and are available under open access (CC BY). For additional information and requests, please contact the lead contact: Charles Winterhalter.

Research involving human participants, their data, or biological material

Policy information about studies with human participants or human data. See also policy information about sex, gender (identity/presentation), and sexual orientation and race, ethnicity and racism.

Reporting on sex and gender	n/a
Reporting on race, ethnicity, or other socially relevant groupings	n/a
Population characteristics	n/a
Recruitment	n/a
Ethics oversight	Faculty of Medical Sciences Ethics route (Newcastle University)

Note that full information on the approval of the study protocol must also be provided in the manuscript.

Field-specific reporting

Please select the one below that is the best fit for your research. If you are not sure, read the appropriate sections before making your selection.

☒ Life sciences ☐ Behavioural & social sciences ☐ Ecological, evolutionary & environmental sciences

For a reference copy of the document with all sections, see nature.com/documents/nr-reporting-summary-flat.pdf

Life sciences study design

All studies must disclose on these points even when the disclosure is negative.

Sample size	All data shown in this study are representative of at least two biological replicates. Technical and biological repeats vary depending on experimental protocols (e.g. single cell microscopy analyses or population-based assays) and are clearly defined in the Methods section, figure legends and Description of Additional Supplementary files.
Data exclusions	No data were excluded.
Replication	All attempts at replication were successful and Source Data is available alongside the manuscript and Supplementary Information.
Randomization	This is not relevant to this study as genetics define groups to compare.
Blinding	Investigators were blinded during all single cell microscopy data analyses.

Reporting for specific materials, systems and methods

We require information from authors about some types of materials, experimental systems and methods used in many studies. Here, indicate whether each material, system or method listed is relevant to your study. If you are not sure if a list item applies to your research, read the appropriate section before selecting a response.

Materials & experimental systems

n/a	Involved in the study	
☐	☒	Antibodies
☒	☐	Eukaryotic cell lines
☒	☐	Palaeontology and archaeology
☐	☒	Animals and other organisms
☒	☐	Clinical data
☒	☐	Dual use research of concern
☒	☐	Plants

Methods

n/a	Involved in the study	
☒	☐	ChIP-seq
☒	☐	Flow cytometry
☒	☐	MRI-based neuroimaging

Antibodies

Antibodies used	Primary antibodies for DnaC, PriA, DnaD, DnaB, DnaI, SSB and FtsZ were produced by Eurogentec.<br>The following commercial antibodies were employed: Dynabeads™ Protein G for Immunoprecipitation (Invitrogen 10009D), anti-Cas9 (Merck SAB4200701), anti-mNeonGreen (ProteinTech 32F6), anti-rabbit (ProteinTech SA00001-2), anti-sheep (Merck A3415), anti-mouse (ProteinTech SA00001-1-A), anti-His (Qiagen 34660), anti-FLAG (Merck M8823).
Validation	Primary antibodies that were purchased from scientific suppliers are suitable for immunoblot/immunoprecipitation and meet all manufacturer's requirements (validation on respective webpages available from the information above).<br>The following primary antibodies have been previously validated/published in peer-reviewed journals: anti-DnaD and anti-DnaB (DOI 10.1093/nar/gkac1060), anti-FtsZ (DOI 10.1093/emboj/19.7.1467).<br>The anti-DnaC, anti-PriA, anti-DnaI and anti-SSB antibodies validation data has been included in the Source Data file.

Animals and other research organisms

Policy information about studies involving animals; ARRIVE guidelines recommended for reporting animal research, and Sex and Gender in Research

Laboratory animals	This study includes research with the model bacterium Bacillus subtilis. Recombinant DNA assembly was performed in vitro or using Escherichia coli as chassis organism.
Wild animals	n/a
Reporting on sex	n/a
Field-collected samples	n/a
Ethics oversight	Faculty of Medical Sciences Ethics route (Newcastle University)

Note that full information on the approval of the study protocol must also be provided in the manuscript.

Plants

Seed stocks	n/a
Novel plant genotypes	n/a
Authentication	n/a
